# Development of a convolutional neural network for diagnosing osteoarthritis, trained with knee radiographs from the ELSA-Brasil Musculoskeletal

**DOI:** 10.1590/0100-3984.2023.0020-en

**Published:** 2023

**Authors:** Júlio Guerra Domingues, Daniella Castro Araujo, Luciana Costa-Silva, Alexei Manso Corrêa Machado, Luciana Andrade Carneiro Machado, Adriano Alonso Veloso, Sandhi Maria Barreto, Rosa Weiss Telles

**Affiliations:** 1 Faculdade de Medicina da Universidade Federal de Minas Gerais (UFMG), Belo Horizonte, MG, Brazil; 2 Instituto de Ciências Exatas da Universidade Federal de Minas Gerais (UFMG), Belo Horizonte, MG, Brazil; 3 Huna-AI, São Paulo, SP, Brazil; 4 Instituto Hermes Pardini, Belo Horizonte, MG, Brazil; 5 Hospital das Clínicas da Universidade Federal de Minas Gerais (UFMG)/Empresa Brasileira de Serviços Hospitalares (EBSERH), Belo Horizonte, MG, Brazil

**Keywords:** Osteoarthritis, knee, Radiography, Neural networks, computer, Machine learning, Diagnosis, computer-assisted, Epidemiologic studies., Osteoartrite do joelho, Radiografia, Redes neurais de computação, Aprendizado de máquina, Diagnóstico por computador, Estudos epidemiológicos.

## Abstract

**Objective:**

To develop a convolutional neural network (CNN) model, trained with the Brazilian “Estudo Longitudinal de Saúde do Adulto Musculoesquelético” (ELSA-Brasil MSK, Longitudinal Study of Adult Health, Musculoskeletal) baseline radiographic examinations, for the automated classification of knee osteoarthritis.

**Materials and Methods:**

This was a cross-sectional study carried out with 5,660 baseline posteroanterior knee radiographs from the ELSA-Brasil MSK database (5,660 baseline posteroanterior knee radiographs). The examinations were interpreted by a radiologist with specific training, and the calibration was as established previously.

**Results:**

The CNN presented an area under the receiver operating characteristic curve of 0.866 (95% CI: 0.842-0.882). The model can be optimized to achieve, not simultaneously, maximum values of 0.907 for accuracy, 0.938 for sensitivity, and 0.994 for specificity.

**Conclusion:**

The proposed CNN can be used as a screening tool, reducing the total number of examinations evaluated by the radiologists of the study, and as a double-reading tool, contributing to the reduction of possible interpretation errors.

## Abstract (pt)

**Objetivo:**

Desenvolver um modelo computacional - rede neural convolucional (RNC) - treinado com radiografias da linha de base do Estudo Longitudinal de Saúde do Adulto Musculoesquelético (ELSA-Brasil Musculoesquelético), para a classificação automática de osteoartrite dos joelhos.

**Materiais e Métodos:**

Trata-se de um estudo transversal abrangendo todos os exames da linha de base do ELSA-Brasil Musculoesquelético (5.660 radiografias dos joelhos em incidência posteroanterior). Os exames foram interpretados por médico radiologista com treinamento específico e calibração previamente publicada.

**Resultados:**

A RNC desenvolvida apresentou área sob a curva característica de operação do receptor de 0,866 (IC 95%: 0,842-0,882). O modelo pode ser calibrado para alcançar, não simultaneamente, valores máximos de 0,907 para acurácia, 0,938 para sensibilidade e 0,994 para especificidade.

**Conclusão:**

A RNC desenvolvida pode ser utilizada como ferramenta de triagem, reduzindo o número total de exames avaliados pelos radiologistas do estudo, e/ou como ferramenta de segunda leitura, contribuindo com a redução de possíveis erros de interpretação.

## INTRODUCTION

Osteoarthritis is one of the most prevalent health problems worldwide, especially in the elderly^**(^1^)**^. Knee osteoarthritis stands out not only for its high prevalence but also for the associated morbidity, being one of the main causes of years lived with disability^**(^2^)**^. In the largest longitudinal study of musculoskeletal disease in Brazil^**(^3^)**^, knee osteoarthritis was identified on radiographs in 18.1% of the participants.

Knee osteoarthritis can cause pain, joint stiffness, reduced range of motion, and muscle weakness^**(^4^)**^. Long-term consequences include a reduction in the level of physical activity and changes in sleep, as well as depression and disability^**(^4^)**^. There are also various economic and social repercussions^**(^5^)**^: the direct costs (of treatments and surgical procedures); the indirect costs (of absenteeism, reduced employability, and early retirement); and the intangible costs (of pain, reduced quality of life, and less social engagement). It is estimated that the total costs related to osteoarthritis can reach 1.0-2.5% of the gross domestic product in developed countries^**(^6^)**^, and there is a tendency for such costs to increase because of the increase in the prevalence of overweight and obesity, as well as because of the aging of the population^**(^5^)**^.

The diagnosis of knee osteoarthritis can be based on clinical criteria, radiographic criteria, or both, the radiographic criteria being considered more sensitive^**(^7^)**^. In longitudinal epidemiological studies, the diagnosis is usually made on the basis of findings from knee radiographs^**(^8^)**^, typically by using the Kellgren and Lawrence (KL) grading system^**(^9^)**^. A KL grade of 0 or 1 indicates the absence of definitive knee osteoarthritis, whereas KL grades 2, 3, and 4 indicate its presence.

The classification of radiographs in longitudinal studies is usually performed by specialist physicians and requires rigorous training, standardization, and calibration^**(^3^)**^. Image analysis consists of semiquantitative grading of osteophytes and joint spaces, according to the radiographic atlas. In large-scale research, this process becomes excessively time-consuming and costly, being subject to the level of experience of the observers. Therefore, studies have been developed with the aim of determining the feasibility of using computational models for automated and semi-automated classification of knee osteoarthritis^**(^10^)**^, in order to reduce the total number of images to be evaluated by humans^**(^11^)**^.

Various artificial intelligence (AI) algorithms have been employed to evaluate medical images. Machine learning is a subfield of AI that includes models that can learn patterns and improve themselves by making comparisons within the database provided^**(^10^,^12^)**^.

**Figure 1:**
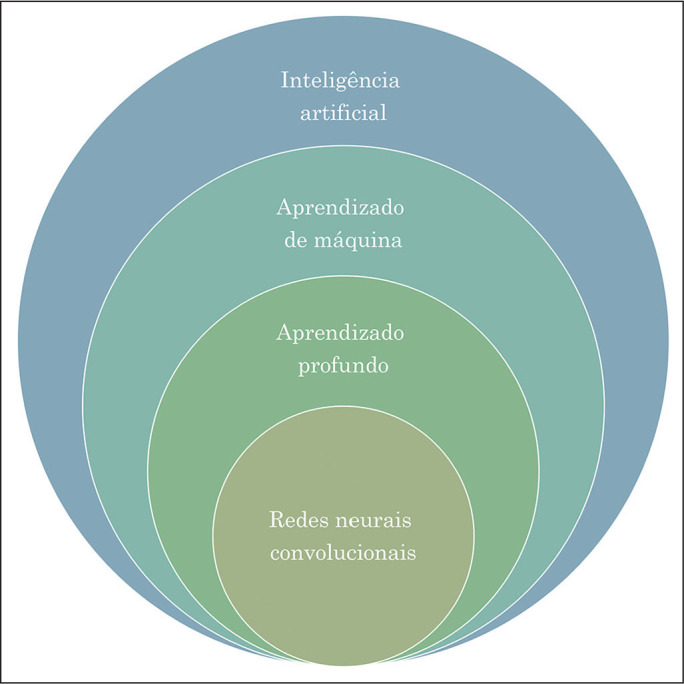
Stacked Venn diagram demonstrating the relationships among the various AI subfields.

Classically, the development of image analysis algorithms has been based on previously selected relevant attributes. However, a more recent machine learning approach, known as deep learning, uses algorithms that identify, by themselves, the characteristics that would best classify data directly from images^**(^11^)**^. Among the deep learning architectures used in the analysis of imaging examinations, convolutional neural networks (CNNs) stand out. In comparison with other AI models, CNNs have demonstrated better performance on that task, especially since 2012, allowing for greater speed and better reproducibility of readings^**(^11^)**^. The relationships among the various AI subfields are illustrated in Figure 1.

In musculoskeletal radiology, a number of studies have investigated the use of AI in tasks such as the diagnosis/classification of fractures, the identification of ligament/meniscal injuries, and the improvement of radiologist workflows^**(^12^)**^.

The training and verification of the accuracy of computational models have been concentrated in clinical and epidemiological studies conducted in the United States^**(^10^,^13^)**^, and tools validated for use in other countries are therefore scarce. Two recent reviews of the topic^**(^10^,^13^)**^ identified no studies that covered the population of Brazil, or even that of Latin America, in the training of the CNNs currently available for the radiographic diagnosis of knee osteoarthritis, thus demonstrating the need for greater external validation.

The *Estudo Longitudinal de Saúde do Adulto* (ELSA-Brasil, Longitudinal Study of Adult Health), the largest longitudinal epidemiological study in Latin America^**(^14^)**^, has, since 2012, included the assessment of musculoskeletal diseases through an ancillary study: the ELSA-Brasil Musculoskeletal (ELSA-Brasil MSK). In addition to the assessments already carried out in the ELSA-Brasil, the ELSA-Brasil MSK incorporates questionnaires on disability/musculoskeletal symptoms, the identification of risk factors for musculoskeletal diseases, and physical performance tests, as well as radiographs of the hands and knees^**(^3^)**^.

The objective of the present study is to propose a computational model for classifying osteoarthritis in knee radiographs, trained with ELSA-Brasil MSK data. The software developed (source code and pre-trained model) is available from the GitHub repository (https://github.com/jgdjulio/kneelsa).

## MATERIALS AND METHODS

### Sample

The development of the computational model for automated analysis of radiographs was carried out on the basis of examinations carried out in the first visit of the ELSA-Brasil MSK cohort. At baseline, the ELSA-Brasil MSK included 2,901 active or retired employees of two large teaching and research institutions in the Brazilian state of Minas Gerais. The mean age of the participants was 56 years (range, 38-79 years), and 52.9% were women. Radiographs of both knees were available for 2,830 of the participants; therefore, images of 5,660 knees were available for analysis. Details about the delineation and profile of the ELSA-Brasil MSK cohort are available elsewhere^**(^3^)**^. The study was approved by the respective research ethics committees of the institutions involved, and participant data are kept confidential at the ELSA-Brasil data center.

### Radiographic examination

Knee radiographs with digital processing were obtained at a radiology clinic affiliated with the ELSA-Brasil, located adjacent to the investigation center. The images were acquired in a bilateral posteroanterior view in fixed flexion, with a patented positioner (patent no. INPI BR102013033625-4) developed by the ELSA-Brasil MSK research team^**(^15^)**^. All examinations were performed by a radiology technician or technologist duly trained and certified in accordance with the study protocol.

The radiographic acquisition protocol was evaluated in a previous study with a test-retest design, demonstrating adequate image quality and reproducibility of quantitative parameters^**(^16^)**^. Other longitudinal studies, such as the Osteoarthritis Initiative (OAI) and the Multicenter Osteoarthritis Study (MOST), have employed similar protocols^**(^17^,^18^)**^.

### Human interpretation

The interpretation of the radiographs was carried out according to the following protocol, as previously published and validated^**(^3^)**^: all examinations were screened for “possible osteoarthritis” by two technologists independently; and all examinations categorized, by at least one of the technologists, as “possible osteoarthritis” were reviewed by a radiologist with specific training. Data regarding agreement between the reading of the ELSA-Brasil MSK radiologist and that of an external reader (a musculoskeletal radiologist with an academic background who was responsible for readings in the Framingham Osteoarthritis Study and MOST), as well as regarding intraobserver agreement for the ELSA-Brasil MSK radiologist, have been published previously^**(^3^)**^. For the radiographic diagnosis of knee osteoarthritis, the interobserver kappa was 0.755 (95% CI: 0.663-0.847) and the intraobserver kappa was 0.891 (95% CI: 0.807-0.975).

Radiographs with a KL grade of 0 or 1 were considered negative for osteoarthritis, whereas those with a KL grade of 2, 3, or 4 were considered positive. A binary classification (osteoarthritis = 0; osteoarthritis = 1) was used as a reference value by the neural network.

### Computational model

Evaluating the most widely used AI techniques for evaluating medical images today^**(^11^,^19^)**^, we highlight CNNs, artificial neural network models composed of interconnected layers (conceptually analogous to biological neurons), which implement a classification process. The first layers detect and extract the primitive attributes of the images (such as edges and texture elements), which are then processed and selected in the subsequent layers. Those attributes are integrated, with different weights, into the output layer, which predicts the class/outcome with the highest probability^**(^11^)**^.

**Figure 2:**
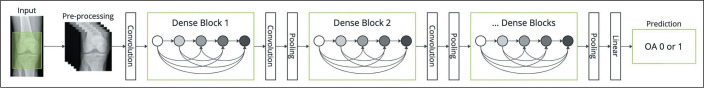
Schematic illustration of the DenseNet architecture. Pairs of layers are connected, allowing elements from the first layers (such as edges) to be used in the subsequent layers as well. Adapted from Huang et al.^(^20^)^.

The CNN model proposed here uses pre-trained 161-layer densely connected architecture, known as a dense convolutional network (DenseNet), as proposed by Huang et al.^**(^20^)**^ and illustrated in Figure 2. In this architecture, subsequent layers also receive information from the initial layers, which avoids the loss of important information (image details) and allows the computational models to be more efficient.

**Figure 3:**
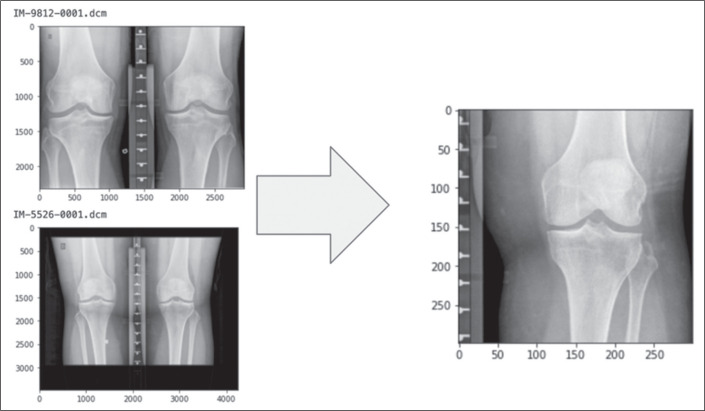
Demonstration of preprocessing.

Images were preprocessed by using raw data from bilateral posteroanterior radiographs of the knees (Digital Imaging and Communications in Medicine files). Initially, the right and left knees were isolated, after which the images were enlarged and resized, in a square matrix, with localization of the regions of interest (femorotibial compartments), as shown in Figure 3.

To increase the number of images available for training the neural network, the following random data augmentation mechanisms were carried out from the torchvision.transforms module of the PyTorch library, applied to the training sample: rotation (0.5°) and Gaussian blur; horizontal inversion; and sharpness adjustment (factor = 0.5) and Gaussian blur. That was followed by resizing, center cropping (CenterCrop function), and normalization. The examinations in the sample were divided into two mutually exclusive subsets (folds): training and testing.

Since the model output is a probability for each image, it can be calibrated by optimizing the thresholds, which range from 0 to 1, a process known as threshold moving. In binary classification problems, the default decision threshold is 0.5: if the probability is greater than this value, it is considered class 1; otherwise, it is considered class 0.

### Data analysis

The binary classifications (osteoarthritis = 0; osteoarthritis = 1) made by the CNN were compared with those of the radiologist (reference values). The performance of the CNN was determined by using the metrics module of the scikit-learn library, version 1.0.2. For each threshold, the proportions of true-positive, true-negative, false-positive, and false-negative results were stored in vectors, from which the mean sensitivity, specificity, precision, accuracy, balanced accuracy, and weighted balanced accuracy, as well as the mean F1 and F2 scores, were calculated for the folds.

Accuracy is calculated by determining the ratio between the number of correct answers (true positives and true negatives) and the total number of examinations evaluated. In unbalanced samples, however, like those evaluated in the present study, in which there are many more examples of normal examinations than altered ones, this metric may not adequately demonstrate the performance of the model. In this context, the use of balanced accuracy allows a better estimate of the CNN yield^**(^21^)**^, being calculated according to the formula: (*sensitivity* + *specificity*) ∕ 2. Some authors also advocate the use of weighted balanced accuracy^**(^21^,^22^)**^, which allows the attribution of different weights to each metric, having been calculated as follows: (2 × *sensitivity* + *specificity*) ∕ 3.

To calculate the area under the receiver operating characteristic curve (AUC) for the model, the predicted probabilities for each image were considered, calculated, and stored in lots of 128 examinations each. Those lots were compared with the true value by using the roc_auc_score function of the scikit-learn library. That function plots the rate of correctly classified positives among all positive predictions (i.e., the true-positive rate) versus incorrect positives among all negatives (i.e., the false-positive rate), at varying thresholds^**(^23^)**^.

## RESULTS

**Figure 4:**
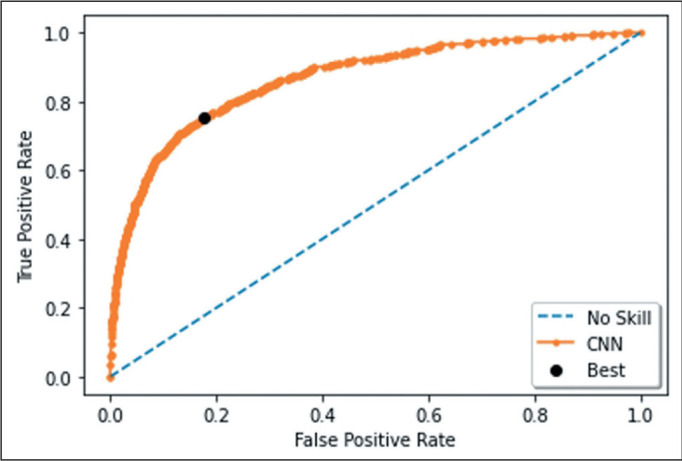
Receiver operating characteristic curve for the model. The black dot demonstrates the threshold of highest balanced accuracy.

Considering the simple average of the two folds, we found that the CNN developed presented an accuracy of 0.814 (at the point of maximum balanced accuracy), with a sensitivity of 0.755 and a specificity of 0.821. As can be seen in Figure 4, the AUC for the model was 0.866 (95% CI: 0.854-0.883).

Following the technique explained above, the model can be calibrated to achieve, not simultaneously, maximum values of 0.907 for accuracy, 0.938 for sensitivity, and 0.994 for specificity. The maximum F1 and F2 scores achieved were 0.553 and 0.619, respectively. Table 1 demonstrates the maximum values achieved by the CNN, according to the optimized metric.

**Table 1:** Accuracy, balanced accuracy, weighted balanced accuracy, sensitivity, specificity, precision, F1 scores, and F2 scores for each defined threshold.

Maximized metric	Threshold	Accuracy	Balanced accuracy	Weighted balanced					
				accuracy	Sensitivity	Specificity	Precision	FI score	F2 score
Sensitivity	0.010	0.400	0.646	0.755	0.973	0.319	0.758	0.272	0.479
Weighted balanced accuracy	0.040	0.649	0.758	0.805	0.899	0.617	0.235	0.373	0.575
Balanced accuracy	0.140	0.814	0.789	0.778	0.756	0.822	0.357	0.485	0.618
Fi score	0.314	0.881	0.774	0.728	0.636	0.913	0.490	0.553	0.600
Accuracy	0.712	0.907	0.671	0.568	0.363	0.978	0.687	0.475	0.401
Specificity	0.900	0.902	0.599	0.467	0.203	0.994	0.806	0.325	0.239

## DISCUSSION

The model developed showed good performance^**(^24^)**^ for the radiographic diagnosis of knee osteoarthritis in the posteroanterior fixed-flexion view. The comparison between the efficiency of different AI models has yet to be standardized in the literature^**(^25^)**^. Despite the fact that there is a historical predilection for accuracy, the AUC is currently considered the most appropriate metric for the assessment of performance^**(^25^)**^.

In the recent major review on the use of machine learning algorithms for the assessment of osteoarthritis, Binvignat et al.^**(^10^)**^ identified only two studies that proposed diagnosing knee osteoarthritis from radiographs alone^**(^26^,^27^)**^. Brahim et al.^**(^26^)**^ achieved 82.98% accuracy (sensitivity: 87.15%; specificity: 80.65%) for differentiating between KL grades 0 and 2 with a decision support tool that was trained on 1,024 images from the OAI, comprising an equal number of grade 0 and grade 2 images. In that study, the AUC was not calculated. The model employed relies, in the segmentation process, on the manual delimitation of bone anatomical landmarks on the tibia, which limits its use in large-scale studies. It would be interesting to include KL grade 1 radiographs and determine the accuracy in a sample with a larger number of patients without osteoarthritis (as occurs in the general population), in order to determine the accuracy of the model in a context approximating that encountered in real life. Tiulpin et al.^**(^27^)**^ created a Siamese neural network for automated KL grading of knee radiographs. The authors used 18,376 MOST radiographs to train the network, 2,957 and 5,960 OAI images being used for validation and testing, respectively. To estimate the performance of the model for diagnosing knee osteoarthritis, they considered KL grade ≥ 2, achieving an AUC of 0.93. During training, serial examinations of participants (from all follow-up visits) and all available X-ray beam angulations (5°, 10°, and 15°) were used, which increased the robustness of the model.

Some techniques to deal with the imbalance between classes have been tried, such techniques including the data augmentation used in the present study, only on positive data, and changing the loss function (increasing by 10 times the penalty for type II errors), neither of which had any effect on the AUC for the model. In fact, recent studies of tabular data^**(^28^)**^ have demonstrated that these and other correction methods can even reduce the AUC, especially for well-performing models^**(^22^)**^.

In the present study, calibration of the neural network through the definition of thresholds was the mechanism that had the greatest impact on the performance metrics. In fact, lowering the threshold for defining knee osteoarthritis increased the sensitivity of the model, whereas raising that threshold increased the specificity.

The model must be calibrated according to the intended application. Therefore, a model with greater balanced accuracy would be more appropriate if its application is as a double-reading tool, whereas a more sensitive model would be preferable for use as a screening method^**(^11^)**^. The same neural network with two or more thresholds, or even more than one neural network, could also be used, especially given the low computational and time costs related to the use of pre-trained models.

Given the specificity achieved by the model, its application is viable in tasks such as checking possible inconsistencies (false negatives) in the database and defining priority in the queue of examinations to be analyzed. Its sensitivity allows its use as a possible screening tool for normal examinations, which would reduce the volume of examinations to be evaluated by radiologists. It is noteworthy that the diagnosis of some diseases, such as knee osteoarthritis, usually requires the radiographic findings to be evaluated in conjunction with clinical, epidemiological, and laboratory data, with or without the findings obtained by other imaging methods, none of which were evaluated in the present study.

Because the CNN employed in our study is a “black-box” model, it is important that its conclusions are based on aspects considered relevant for the diagnosis, in a way that is understandable to humans^**(^29^)**^. This factor, known as the explainability or interpretability of the network, can be expressed in the form of attention maps, which highlight the regions of the image most related to the prediction made by the model (e.g., osteophytes, joint spaces, and sclerosis). Explainability tools for the CNN employed here are still under development, which constitutes a current limitation of the model.

The training and validation of the CNN were based on the interpretation of two technologists and a radiologist, in accordance with the ELSA-Brasil MSK radiograph classification workflow and in compliance with strict quality control guidelines^**(^3^)**^. However, the inclusion of examinations from other longitudinal studies, with reports composed by a committee of radiologists, could increase the robustness of the network, representing a future step in its development. Nevertheless, given the accuracy achieved, we can conclude that the model was able to learn how to interpret knee radiographs.

The ELSA-Brasil MSK radiographs were obtained by trained technologists, in a standardized manner, in a specific view, and using a positioner suitable for evaluating knee osteoarthritis. However, the view most often used in medical practice, despite being less accurate for assessing knee osteoarthritis, is the anteroposterior view with knee extension^**(^8^)**^, and it is therefore not possible to extrapolate our results to outpatient or hospital settings in general. In addition, only one view (posteroanterior fixed-flexion) was employed to develop the CNN employed in our study. However, only 9.9% of individuals with radiographically confirmed knee osteoarthritis in the ELSA-Brasil MSK had isolated osteoarthritis identified on the lateral view^**(^3^)**^. The performance of this CNN has yet to be tested in populations from other studies (such as the OAI and MOST).

Another limitation is the reduction in image resolution during preprocessing, which is common in the development of AI models. Despite enabling greater time-effectiveness, this mechanism can limit the results because of the loss of subtle information from the examinations.

Improvements to the model presented, using images from subsequent waves of the ELSA-Brasil MSK, as well as image databases from other studies (such as the OAI and MOST), should contribute to increasing the performance and robustness of the network. Such improvements are being implemented and may be addressed in future works.

## CONCLUSIONS

The CNN developed presents performance comparable to that of neural networks trained with radiographs from international studies. The accuracy and AUC achieved allow its use as a double-reading tool in the ELSA-Brasil MSK, helping overcome the problem of the limited availability of trained radiologists, as well as reducing the costs of and time spent on interpreting knee radiographs.

Validation of the model in populations different from the one in which it was trained, in other longitudinal studies and in clinical practice, is important for its future adoption. Therefore, we reiterate that the software developed is publicly available in the GitHub repository (https://github.com/jgdjulio/kneelsa), which makes its external validation possible in future studies.
